# 9-PAHSA Prevents Mitochondrial Dysfunction and Increases the Viability of Steatotic Hepatocytes

**DOI:** 10.3390/ijms21218279

**Published:** 2020-11-05

**Authors:** Adriana R. Schultz Moreira, Sabrina Rüschenbaum, Stefan Schefczyk, Ulrike Hendgen-Cotta, Tienush Rassaf, Ruth Broering, Matthias Hardtke-Wolenski, Laura Elisa Buitrago-Molina

**Affiliations:** 1 Department of Gastroenterology and Hepatology, University Hospital Essen, University Duisburg-Essen, 45147 Essen, Germany; adriana.schultz-moreira@uk-essen.de (A.R.S.M.); sabrina.rueschenbaum@uk-essen.de (S.R.); Stefan.Schefczyk@uk-essen.de (S.S.); Ruth.Broering@uk-essen.de (R.B.); Matthias.Hardtke-Wolenski@uk-essen.de (M.H.-W.); 2 West German Heart and Vascular Center, Department of Cardiology and Vascular Medicine, University Hospital Essen, University of Duisburg-Essen, 45147 Essen, Germany; ulrike.hendgen-cotta@uk-essen.de (U.H.-C.); Tienush.Rassaf@uk-essen.de (T.R.); 3 Department of Gastroenterology, Hepatology & Endocrinology, Hannover Medical School, 30625 Hannover, Germany

**Keywords:** 9-PAHSA, steatosis, oleic acid, cell viability, primary murine hepatocytes, oil red O, lipids

## Abstract

Nonalcoholic fatty liver disease (NAFLD) is quickly becoming the most common liver disease worldwide. Within the NAFLD spectrum, patients with nonalcoholic steatohepatitis (NASH) are at the highest risk of developing cirrhosis and disease progression to hepatocellular carcinoma. To date, therapeutic options for NASH patients have been ineffective, and therefore, new options are urgently needed. Hence, a model system to develop new therapeutic interventions is needed. Here, we introduce two new in vitro models of steatosis induction in HepG2 cells and primary murine hepatocytes. We used a recently discovered novel class of bioactive anti-inflammatory lipids called branched fatty acid esters of hydroxyl fatty acids. Among these bioactive lipids, palmitic-acid-9-hydroxy-stearic-acid (9-PAHSA) is the most promising as a representative nondrug therapy based on dietary supplements or nutritional modifications. In this study, we show a therapeutic effect of 9-PAHSA on lipotoxicity in steatotic primary hepatocytes and HepG2 cells. This could be shown be increased viability and decreased steatosis. Furthermore, we could demonstrate a preventive effect in HepG2 cells. The outcome of 9-PAHSA administration is both preventative and therapeutically effective for hepatocytes with limited damage. In conclusion, bioactive lipids like 9-PAHSA offer new hope for prevention or treatment in patients with fatty liver and steatosis.

## 1. Introduction

Nonalcoholic fatty liver disease (NAFLD) is one of the most common liver diseases worldwide. NAFLD affects approximately one-quarter of the world’s adult population, and its incidence is rapidly increasing [1]. The NAFLD disease spectrum includes nonalcoholic fatty liver (NAFL) and the advanced form of nonalcoholic steatohepatitis with inflammation and mitochondrial dysfunction (NASH) [2]; both of these conditions are characterized by the accumulation of fat in more than 5% of hepatocytes [3,4]. In addition to steatosis, hallmarks of NASH include hepatocyte injury and inflammation. Further progression to end-stage liver cirrhosis is possible and common. Moreover, NASH is a significant risk factor for the development of hepatocellular carcinoma [5,6].

Therapeutic options for NASH patients are still limited and are therefore urgently needed. Weight loss, healthy food choices and physical activity are the only established therapeutic strategies established thus far, as they also attenuate the underlying metabolic dysfunction [7]. Moreover, most of the current trials are mainly focused on hepatic improvement. To our knowledge, no investigation has considered using bioactive lipids in a NASH experimental model. In this study, we suggest a nondrug therapy based on dietary supplements or nutritional modifications.

A novel class of bioactive lipids called branched fatty acid esters of hydroxyl fatty acids (FAHFAs) was recently discovered [8]. FAHFA families are characterized by distinct fatty acid and hydroxy fatty acid compositions [8]. Intestinal permeability plays an important role in the progression of NASH development [9]. Interestingly, Kahn’s group demonstrated that FAHFAs regulate innate and adaptive immune responses to prevent mucosal damage [10]. Palmitic-acid-9-hydroxy-stearic-acid (9-PAHSA) is an isomeric form of a bioactive FAHFA [8]. In serum from insulin-resistant patients and insulin-sensitive mice, the endogenous 9-PAHSA level was found to be decreased [8] and increased [11], respectively. Additionally, 9-PAHSA exhibited anti-inflammatory properties [8]. The positive correlation observed between 9-PAHSA levels and the regulation of systemic glucose homeostasis encouraged further studies of FAHFAs in the context of metabolic disorders.

In the present study, we show two important results. First, we show alternative ways to implement the 3R principle in NASH research by establishing a new complementary model of in vitro steatotic hepatocytes. Second, our research led to the development of a nondrug therapy based on dietary supplements or nutritional modifications. We showed the therapeutic effect of 9-PAHSA against lipotoxicity on steatotic hepatocytes and a preventive effect on HepG2 cells. The outcome of 9-PAHSA administration was both preventative and therapeutically effective in hepatocytes with limited damage.

## 2. Results

### 2.1. Pretreatment with 9-PAHSA Prevented Mitochondrial Dysfunction in Steatotic Cells

**Figure 1:**
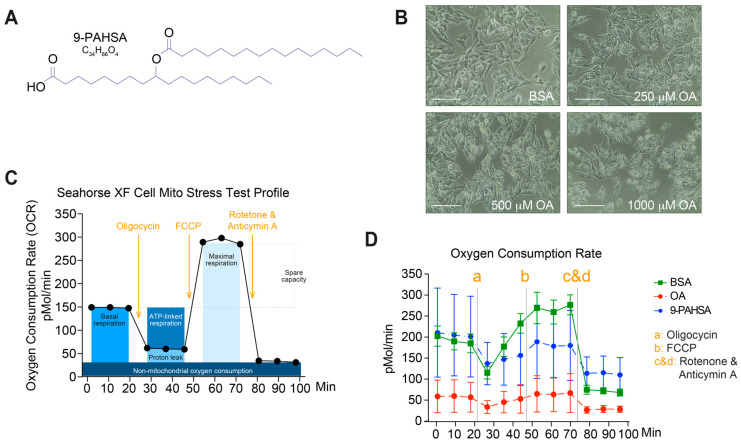
Pretreatment with 9-PAHSA prevented mitochondrial dysfunction in the steatotic cells. (**A**) Chemical structure and molecular formula of 9-PAHSA. B–D Steatosis was induced using OA in HepG2 cells followed treatment with 9-PAHSA. (**B**) Representative pictures of OA-induced steatosis in HepG2 cells and non-induced control. Indicated OA concentrations were used. Magnification bar represents 100 μm. (**C**) Seahorse XF Cell Mito Stress Test profile indicating the key parameter obtained in one assay. (**D**) Mitochondrial respiration was determined for the 12 h 9-PAHSA-treated HepG2 cells and controls followed by 6 h 500 μM OA. Points represent the means ± SEM.

Endogenous 9-PAHSA has been shown to have a role in insulin sensitivity and as an anti-inflammatory and antioxidant agent [8,11]. In this study, we were interested in elucidating the preventive effects of 9-PAHSA (Figure 1A) on steatosis in vitro.

Therefore, HepG2 cells were incubated with 9-PAHSA for 12 h and then exposed to oleic acid (OA) for 6 h to induce steatosis. Bovine serum albumin (BSA) was used as a control (Figure 1B). Proper mitochondrial function is critical for organ homeostasis. Several studies corroborated that mitochondrial dysfunction plays a role in NASH development [2,12]. Using a Seahorse Extracellular Flux Analyzer, we examined the cellular oxygen consumption rate in OA-treated and control living HepG2 cells as a measure of mitochondrial respiration. The instrument continuously measures in real-time the oxygen concentration in the cell culture supernatant over time and detects the effects of respiration modulators, revealing key parameters of mitochondrial function: oligomycin inhibits ATP synthase, carbonyl cyanide-4 (trifluoromethoxy) phenylhydrazone (FCCP) disrupts mitochondrial membrane potential and rotenone (complex I inhibitor) and antimycin A (complex III inhibitor) stop mitochondrial respiration. The measurements obtained for these parameters with this assay are shown in Figure 1C. We observed that steatotic cells displayed impaired mitochondrial respiration, as determined by the measurement of oxygen consumption rate (OCR). The toxic effects of OA were demonstrated by a decrease in nonmitochondrial respiration, ATP-linked respiration and maximal respiration. FCCP injection, which stimulates maximal respiratory capacity, had only a small effect, indicating that upon exposure to OA, HepG2 cells undergo profound mitochondrial dysfunction. In agreement with our hypothesis, pretreatment with 9-PAHSA prevented and recovered the mitochondrial dysfunction observed in steatotic cells. As expected, BSA controls exhibited a normal mitochondrial respiration profile. (Figure 1D).

### 2.2. 9-PAHSA Treatment Improved the Viability of the Steatotic Primary Murine Hepatocytes (PMH)

**Figure 2:**
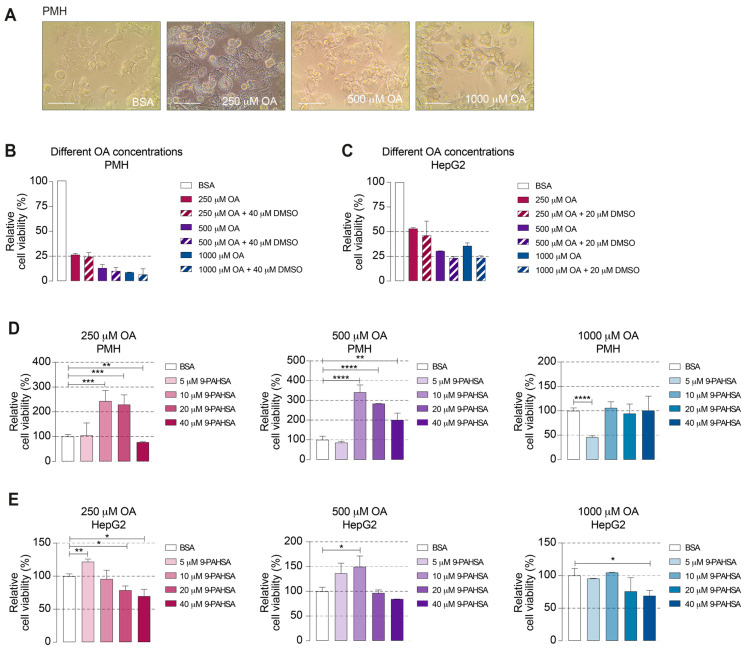
Treatment with 9-PAHSA increased the viability of the steatotic primary murine hepatocytes (PMH) and HepG2 cells. (**A**–**E**) PMH and human HepG2 cells were challenged with OA to induce steatosis and treated with different concentrations of 9-PAHSA. (**A**) Representative pictures of OA-induced steatosis in PMH and non-induced control. Magnification bar represents 100 μm. (**B**,**C**) Viability index relative to bovine serum albumin (BSA) control of steatotic PMH (**B**) and HepG2 cells (**C**) induced with indicated OA concentrations. Bars display cell viability after OA treatment as assessed by WST-1 assay. (**D**) Viability index of PMH or (**E**) HepG2 cells induced with indicated concentrations of OA and treated different 9-PAHSA concentrations. All bars represent the means ± SEM. * *p* ≤ 0.05, ** *p* ≤ 0.01, *** *p* ≤ 0.001 and **** *p* ≤ 0.0001.

We demonstrated that 9-PAHSA had a protective effect on mitochondrial respiration following OA incubation. Thus, we wondered whether 9-PAHSA could be used as a therapeutic agent for steatosis. To evaluate this possibility, we isolated hepatocytes from 8-week-old C57BL/6 mice and used as before HepG2 cells. The hepatic cells were incubated with three different concentrations of OA for 24 h to induce steatosis (Figure 2A).

The WST-1 cell proliferation assay showed a significant concentration-dependent reduction in the viability of the OA-treated primary murine hepatocytes (PMH) and HepG2 cells. Exposure to DMSO as a diluent agent for 9-PAHSA did not alter the viability of the cells (Figure 2B,C). Subsequently, to investigate the effects of 9-PAHSA in steatotic PMH and HepG2 cells, hepatic cells were incubated with four different concentrations of 9-PAHSA for 6 h (Figure 2D,E). PMH treated with 250 µM and 500 µM OA that were exposed to 10 µM or 20 µM 9-PAHSA, showed increased viability, up to 144% and 240%, respectively, compared to the controls (Figure 2D). At the highest concentration, 9-PAHSA increased the viability only of the cells treated with 500 µM OA, indicating that the 9-PAHSA-rescuing effect is associated with preexisting cell damage. Lower 9-PAHSA concentrations did not lead to a positive outcome in this setup. The viability of the PMH exposed to higher OA concentrations was less than 10% and was not recovered by 9-PAHSA (Figure 2B,D). On the other hand, concentrations of 9-PAHSA that were excessively high were similarly ineffectual or even induced toxicity, as indicated by the viability of the PMH treated with 250 µM OA diminishing with a 40 µM 9-PAHSA treatment (Figure 2C,D). In HepG2 cells, lower 9-PAHSA concentrations improved cell viability in steatotic cells treated with 250 µM and 500 µM OA up to 21% and 32%, respectively. Additionally, 20 µM 9-PAHSA in 500 µM OA-treated cells showed the largest increase in viability with 50% when compared to the controls. As seen in PMH, higher 9-PAHSA concentrations significantly reduced the viability displaying a toxic effect. These suggest that 9-PAHSA had a stronger rescuing effect in PMH than in HepG2 cells. Taken together, these data revealed that 9-PAHSA increased the viability of steatotic PMH and HepG2 cells. This rescuing effect depended on the degree of preexisting damage and 9-PAHSA concentration.

### 2.3. 9-PAHSA Treatment Reduced the Intracellular Lipid Accumulation in Steatotic Cells

The effect that the viability of PMH and HepG2 with steatosis could be improved by the administration of 9-PAHSA is remarkable. However, the mechanisms behind remain still unclear. Among other things, our suggestion would be a direct effect on the lipid accumulation in the cells. Therefore, we introduced here a method to quantify the amount of Oil Red O incorporated by OA-treated hepatic cells.

**Figure 3:**
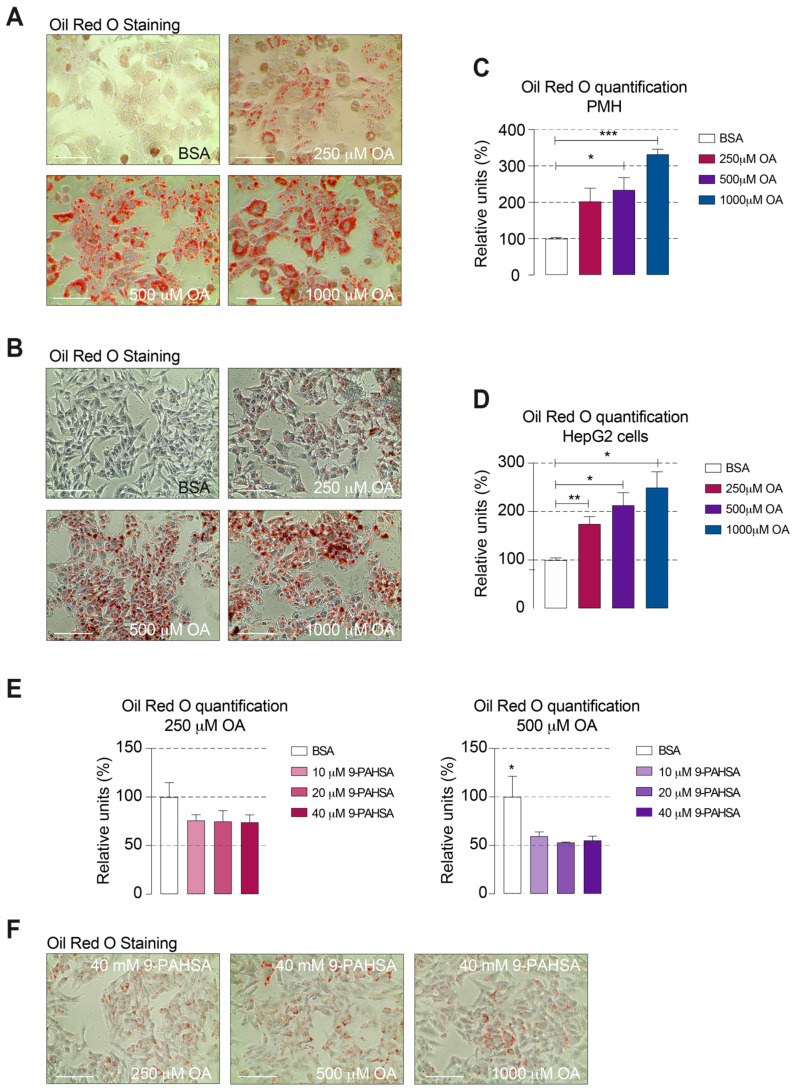
9-PAHSA treatment reduced the intracellular lipid accumulation in HepG2 cells. Representative pictures of Oil Red O staining after OA-induced steatosis in PMH (**A**) or in HepG2 cells (**B**). Quantification of intracellular lipid content in PMH (**C**) or HepG2 cells (**D**). (**E**) Quantification of intracellular lipid content in HepG2 cells after 9-PAHSA treatment with the indicated concentrations. (**F**) Representative pictures of Oil Red O staining after OA-induced steatosis in HepG2 cells followed by treatment with 40 µM 9-PAHSA. Magnification bar represents 100 μm. All bars represent the means ± SEM. * *p* ≤ 0.05, ** *p* ≤ 0.01 and *** *p* ≤ 0.001.

To prove the steatotic induction in PMH and HepG2 cells, we used Oil Red O staining. Steatotic cells exhibited a strong staining showing the intracellular lipid accumulation (Figure 3A,B) Additionally, we could nicely show a positive proportional correlation between the amount of OA for steatosis induction and the amount of incorporated Oil Red O (Figure 3C,D). Since we have shown that the induction with 250 µM and 500 µM kept the PMH and HepG2 cells viable, we used these concentrations for further 9-PAHSA treatment studies in HepG2. 9-PAHSA was used in the effective concentrations of 10 µM, 20 µM and 40 µM in comparison to untreated steatotic control cells (Figure 3E). The direct positive effect of 9-PAHSA on a reduction in steatosis associated with increased viability was clearly demonstrated. In line with our hypothesis, incubation with higher OA concentrations result in a stronger steatosis in PMH and HepG2 cells. Furthermore, exposure to 9-PAHSA reduced the intracellular lipid accumulated in OA-treated HepG2 cells (Figure 3F).

## 3. Discussion

In this paper, we reported two new in vitro models for the complementary study of NASH by inducing hepatic steatosis with OA, which is a fat-carrying agent. In addition, we tested, for the first time, 9-PAHSA in an experimental NASH model. These in vitro methods support the 3R principle used in animal experimentation: an alternative to animal experiments (replace), use of the absolute minimum number of animals (reduce) and ensure minimal animal stress (refine). With these models, we showed the positive effects of 9-PAHSA used for both prevention and treatment in NASH-like PMH and HepG2 cells, which can be used as substitutes for primary hepatocytes. Additional research in other NASH diet models would complement our results and investigation.

The role of 9-PAHSA as an antidiabetic and anti-inflammatory agent is controversial. Increased and decreased levels of endogenous PAHSAs in serum have been found in insulin-resistant patients and insulin-sensitive mice, respectively [8,11]. PAHSA levels are highly correlated with insulin sensitivity in humans and are regulated by different mechanisms [8]. In murine models of insulin resistance, colitis and type 1 diabetes, the anti-inflammatory effects of 9-PAHSA have been previously shown [8,10,13,14,15]. Enhanced glucose tolerance and insulin sensitivity in both chow-fed and high fat diet (HFD)-fed mice were observed after PAHSA administration; however, these beneficial metabolic effects were more modest in the HFD-fed mice [13], suggesting that PAHSAs have a more powerful effect as preventative measures than as treatments. In addition, PAHSA administration also attenuated adipose tissue inflammation in the chow-fed mice [13], recommending its use in NASH prevention for patients at risk.

Nevertheless, Pflimlin et al. using a murine model of diet-induced obesity reported no detection of antidiabetic effects after PAHSA treatment [16]. These contrary studies may result in large part to the methodology chosen [17], suggesting issues in studying PAHSAs. For example, the vehicle utilized to formulate the PAHSA is critical not only to assure bioavailability and safety but also to avoid masking the effects of PAHSA. In NASH, in addition to metabolic disturbance, the accumulation of lipids in hepatocytes, oxidative stress and decreased ATP production trigger mitochondrial dysfunction [18]. In the current work, we showed an additional protective function of 9-PAHSA in an in vitro model of steatosis. Pretreatment with 9-PAHSA prevented the HepG2 hepatic cells from mitochondrial dysfunction and damage caused by high lipid concentrations.

FAHFAs are bioactive lipids present not only in mammalian tissues but also in a diverse range of foods. Clementine, whole grain oat, garlic, pineapple and avocado are examples of food with the highest number of FAHFA families. The most studied group of FAHFAs, PAHSAs, are mostly found in carrot, mango, banana, whole grain oat and apple [19]. In particular, 9-PAHSA is found in broccoli, beef and egg white [8]. The inclusion of foods high in PAHSAs can be a good strategy to help at-risk patients in the prevention of tissue inflammation, steatosis and the development of NASH.

Using PMH, we showed that OA could be used as a fat-carrying agent to mimic NASH in vitro. The viability of the NASH-like cells was dramatically reduced in an OA-concentration-dependent manner. The capacity of OA in inducing steatosis was previously shown in rat primary hepatocytes [20]. Although Moravcova et al. reported a decrease in cell viability, this effect was not as severe as we show here. At an OA concentration of 1000 µM, they described a reduction of less than 40% compared to the control. We observed that only 250 µM OA reduced hepatocyte viability, up to 25%, compared to that of the controls 24 h after treatment. However, comparisons are difficult to make due to differences in the donor animals.

After the induction of steatosis with OA, treatment with 9-PAHSA led to increased cell viability that was concentration dependent. While low 9-PAHSA concentrations seemed to have no effect on cell toxicity, high concentrations increased it. To our knowledge, no studies have dealt with PAHSA overdose. This bioactive lipid is produced endogenously in mammals and exists in food in a small amount. This suggests that the risk of toxicity due to ingested food is low. However, it has been established that even most essential vitamins and minerals have negative health effects when overdosed [21].

A recent publication reported that 9-PAHSA treatment increases islet β cell viability during cytokine-induced cell death. This outcome was due to PAHSAs attenuating ER stress [14], and this mechanism explains the increased viability of steatotic hepatocytes upon 9-PAHSA treatment. However, when steatotic PMH are severely damaged, the effect of 9-PAHSA disappears and cannot attenuate OA-induced toxicity. Mice fed a high-fat diet (60%) receiving acute 9-PAHSA treatment showed no improvement in glucose tolerance [16]. This finding suggested that the enhancement effects of 9-PAHSA depend on pre-established cell damage, a finding in line with our data.

To elucidate the 9-PAHSA impact on hepatosteatosis and NASH, additional investigation is required. Utilizing human hepatocytes and generating organoids would be advantageous in these studies.

The growing epidemic of obesity and fatty liver requires new strategies for prevention and treatment. Our data revealed that 9-PAHSA could be used in steatotic liver. The inclusion of 9-PAHSA-containing food in the diet may help in the prevention of NASH in patients at risk. Considering the hypoglycemic and anti-inflammatory roles of 9-PAHSA, its incorporation into the diet can additionally benefit chronic diseases such as obesity, metabolic syndrome and diabetes. Since 9-PAHSA treatment cannot rescue severely damaged cells, an alternative is to develop synthetic products and incorporate them into practical diet formulations as supplemental aids in the treatment of NASH.

## 4. Materials and Methods

### 4.1. Ethics Statement

Animal care and experiments were performed in accordance with institutional and national guidelines. All animal experiments were performed according to protocols approved by the Animal Welfare Commission of University Hospital Essen (Essen, Germany) and the local Ethics Animal Review Board (Landesamt für Natur, Umwelt und Verbraucherschutz NRW, LANUV). The procedure for mouse hepatocyte preparation has been adapted to the use dead animals sacrificed by cervical dislocation. Under LANUV’s regulations, no additional applications are required to perform that technique. *C57Bl/6J* mice were maintained under specific pathogen-free conditions at the Central Animal Facility of University Hospital Essen (Essen, Germany).

### 4.2. Primary Murine Hepatocyte Purification

PMH were prepared from eight-week-old *C57Bl/6J* mice by collagenase perfusion as described previously [22].

### 4.3. Steatosis Induction

Oleic acid (Merck/ Sigma-Aldrich, Darmstadt, Germany) stock solution was complexed to BSA at a 5:1 molar ratio. For exposure to OA, culture medium supplemented with fetal bovine serum (10% *v/v*) was replaced with fatty acid-free BSA (1% *v/v*). The medium for culturing the PMH was replaced with fresh medium containing 250 μM, 500 μM, or 1000 μM OA and incubated with cells for 24 h. For HepG2 cells, the medium was replaced by 500 μM oleic acid for six hours after 9-PAHSA treatment.

### 4.4. Red Oil O Staining and Quantification

OA-treated PMH and HepG2 cells were fixed with formalin 10% for 1 h. After rinsing steps, cells were consecutively incubated with 60% 2-propanol for 5 min and with Oil Red O working solution (Merck/ Sigma-Aldrich, Darmstadt, Germany) for 20 min. Cells were rinsed until no excess stain was seen. Cells were incubated with hematoxylin for 1 min followed by rinsing steps. They were covered with distilled water and visualized under a microscope. For dye elution, an incubation with 100% 2-propanol for 10 min was performed. Absorbance was measured at 492 nm with a spectrophotometer.

For HepG2 cells, intracellular lipid accumulation was additionally visualized after 6 h 9-PAHSA treatment and measured by absorbance at 492 nm.

### 4.5. 9-PAHSA Treatment

9-PAHSA (Merck/ Sigma-Aldrich, Darmstadt, Germany) was dissolved in 100% DMSO to a final stock concentration of 100 mM. For the PMH, the medium for steatosis induction was replaced with fresh medium containing 5 μM, 10 μM, 20 μM or 40 μM 9-PAHSA for six hours. For the prevention experiments with HepG2 cells, medium containing 9-PAHSA was added to the cells, which were cultured for 12 h before steatosis induction.

### 4.6. Cell Viability

PMH were cultured overnight in 96-well plates at a density of 5 × 10^4^ cells/well. After steatosis induction and 9-PAHSA treatment, cell viability was measured using a cell proliferation reagent WST-1 kit (Merck/ Roche, Darmstadt, Germany) according to the manufacturer’s instructions. The absorbance was measured by a spectrophotometer (FLUOstar Omega, Ortenberg, Germany).

### 4.7. Mitochondrial Respiration

HepG2 cells were plated overnight on collagen-coated, XF 24-well cell culture microplate at a density of 4 × 10^4^ cells/100 μL medium/well. After pretreatment with 9-PAHSA, the culture medium was replaced with fresh medium containing OA.

Mitochondrial OCR was assessed with a Seahorse XF24 Analyzer. The measurements were performed according to the manufacturer’s instructions. The following concentrations were used: 1 μM oligomycin, 0.25 μM FCCP and a mix of 0.5 μM rotenone and 0.5 μM antimycin A. Agilent Seahorse Wave Desktop software, Wave 2.6.1, for the assay design and data analysis (https://www.agilent.com/en/product/cell-analysis/real-time-cell-metabolic-analysis/xf-software/seahorse-wave-desktop-software-740897) was used. Data are expressed as mean ± SEM from 3 technical replicates.

### 4.8. Statistical Analysis

Prior analysis, WST-1 and Oil Red O data were normalized to their corresponding controls. The unpaired Student’s two-tailed *t*-test analyses were performed using Prism 8 software (GraphPad). Data are presented as the mean ± standard error of the mean.

## Abbreviations

NAFLD	Nonalcoholic fatty liver disease
NASH	Nonalcoholic steatohepatitis
9-PAHSA	Palmitic-acid-9-hydroxy-stearic-acid
NAFL	Nonalcoholic fatty liver
FAHFA	Branched fatty acid esters of hydroxyl fatty acid
OA	Oleic acid
FCCP	Phenylhydrazone
OCR	Oxygen consumption rate
HFD	High fat diet
BSA	Bovine serum albumin
